# Motivation to participate in structured physical activity for autistic youth: A systematic scoping review

**DOI:** 10.1177/13623613241240603

**Published:** 2024-04-04

**Authors:** Michelle L Wong, Sonya Girdler, Bahareh Afsharnejad, Nikos Ntoumanis, Ben Milbourn, Paul Kebble, Susan Morris, Melissa H Black

**Affiliations:** 1 Curtin School of Allied Health, Curtin University, Perth, Western Australia; 2 Curtin Autism Research Group, Curtin University, Perth, Western Australia; 3 Centre of Neurodevelopmental Disorders (KIND), Department of Women’s and Children’s Health, Centre for Psychiatry Research, Karolinska Institute and Region Stockholm, Sweden; 4 Curtin School of Population Health, Curtin University, Perth, Western Australia; 5 Danish Centre for Motivation and Behaviour Science, Department of Sports Science and Clinical Biomechanics, University of Southern Denmark, Denmark; 6 Norwegian School of Sport Sciences, Oslo, Norway; 7 Office of the Pro-Vice Chancellor, Health Sciences, Curitn University, Perth, Western Australia

**Keywords:** adolescents, autism, autism spectrum disorders, environmental factors, motivation, physical activity, school-age children, self-determination theory, youth

## Abstract

Autistic youth participate in less mainstream physical activity than their neurotypical peers. A scoping review was conducted to explore motivational factors influencing participation in structured physical activity for autistic youth. Relevant databases were searched using the Preferred Reporting Items for Systematic Reviews and Meta-Analyses with the inclusion of stakeholder consultation, to synthesise existing literature describing the participation and motivations of autistic youth in structured physical activities. Eighteen publications met the eligibility criteria. Qualitative data were extracted and analysed using directed content analysis and then mapped to the motivational constructs of the self-determination theory. Autism-specific themes falling outside of the self-determination theory were coded inductively. Competence was the most reported psychological need (*k* = 14), with intrinsic motivation being the most common motivational regulator (*k* = 12). Inductive analysis revealed one additional theme, which was the impact of the sensory environment on autistic youths’ motivation to participate. Findings indicate that meeting the psychological needs of autistic youth support self-determined motivation to participate in structured physical activities, although fulfilling these needs may differ from their neurotypical peers. Additional autism-specific factors may also influence motivation to participate in structured physical activities. Future research should examine motivational factors that support engagement in structured physical activities through the lens of autistic youth and their experiences.

**Lay abstract:**

Autistic youth participate less in physical education classes and organised sport than their neurotypical peers. We conducted a review of existing studies to investigate what is known about what motivates (and does not motivate) autistic youth to take part in structured physical activities. We systematically searched electronic databases and found 18 publications that met the criteria to be included in this review. Data from these studies were extracted and mapped to the self-determination theory to identify factors that support (or undermine) motivation for autistic youth. We also discussed the findings with autistic individuals and other relevant stakeholders to discover how the review related to their experiences. Our results found competence (youth feeling competent in their athletic and social skills and abilities) to be the most reported psychological need impacting motivation for autistic youth. Intrinsic motivation (participating for enjoyment and satisfaction) was the most common facilitator of motivation. Autism-specific themes outside of the self-determination theory were mapped inductively, and we found that the sensory environment was a prominent theme reported to influence the motivation of autistic youth not covered by the self-determination theory. The findings of this review suggest that supporting the psychological needs of autistic youth can foster motivation to engage in physical activity, although how these needs are met can differ from their neurotypical peers. Future research should examine motivational factors that support engagement in structured physical activities through the lens of autistic youth and their experiences.

Despite the widely recognised benefits of physical activity (PA) for children and adolescents, only 26% of Australian children aged 5–12 years and 8% of adolescents aged 13–17 years meet the PA guidelines of engaging in 60 minutes of moderate to vigorous exercise per day (Australian Institute of Health and Welfare, 2018). These trends are observed internationally, with global estimates indicating that 75% of neurotypical adolescents are not meeting their daily PA requirements (Bull et al., 2020). Broadly, PA is defined as any bodily movement resulting in energy expenditure (Caspersen et al., 1985) and can be divided into two types, structured physical activity (SPA) and unstructured PA. While unstructured PA can be synonymous with unstructured or self-selected free play (e.g. playing on a playground), SPA is PA that is planned, structured and repetitive and often goal directed (Caspersen et al., 1985), for example, school physical education (PE), organised club sports or group exercise classes. In Australia, SPA is typically how youth obtain their PA (Schranz et al., 2018).

Although engagement in PA is low for all Australian children and adolescents, autistic youth participate at a lower rate than their neurotypical peers (Edwards et al., 2017; Pan et al., 2021). The lower participation in PA by autistic youth is of concern given that reduced PA can negatively influence the mental and physical health and well-being of children and adolescents (Bessa et al., 2019; Mygind et al., 2019; Spruit et al., 2016). Previous research suggests that autistic youth spend as little as 17 minutes in vigorous PA per day, which further declines into adulthood (Jones et al., 2017; MacDonald et al., 2011). Autistic youth spend less time engaging in mainstream PE classes than their neurotypical peers (Healy et al., 2013; Pan et al., 2011) and have poorer participation rates in PA outside of the school environment (Jachyra et al., 2021; Ryan et al., 2018), resulting in less opportunity for this population to reach the recommended PA requirements.

Several personal and environmental factors have been identified as contributing to the low participation rates of autistic youth in PA. Factors associated with the core diagnostic criteria of autism, including differences in social communication and interaction, altered sensory processing and the presence of restricted and repetitive patterns of behaviour or interests (American Psychiatric Association [APA], 2013), have been recognised as potential barriers to participation for autistic youth (Case et al., 2020; Pan et al., 2021).

Differences in the social communication and interaction between autistic and non-autistic neurotypes can mean that instructions and social cues are difficult to understand (Healy et al., 2013), while variations in temperature, noise and tactile stimulation can cause discomfort due to altered sensory processing (Arnell et al., 2018; Healy et al., 2013; Yessick et al., 2020). A preference for routine and predictability (Schaaf et al., 2011) can also negatively impact autistic youths’ motivation to participate in PA if the routine changes or there is no routine in place (Adams et al., 2018). Furthermore, while not part of the diagnostic criteria, it is estimated that 35% of autistic individuals experience significant motor difficulties (Licari et al., 2020), which can additionally inhibit participation in mainstream PA. Autistic youth are also more likely to be excluded and bullied during PA than their non-autistic peers and face negative stigma, further limiting their engagement (Healy et al., 2013; Jachyra et al., 2021). The complexities of participating in PA for autistic youth have been recognised by Arnell et al. (2018) in the conditional participation model, which highlights the role of freedom of choice, competence and confidence, predictability, adjustment to external demands and motivation in facilitating the participation of autistic youth in PA (Arnell et al., 2018).

Despite motivation being identified as a factor influencing the participation of autistic youth in PA (Arnell et al., 2018), limited research has investigated the potential mechanisms underlying autistic youths’ motivation to participate in PA. Broadly applied, motivation is an umbrella term describing internal and external factors influencing an individual’s decision and willingness to participate in an activity (Gaudreau & Antl, 2008). Quantitative studies suggest that autistic adolescents have lower motivation to participate in PE than their neurotypical peers (Pan et al., 2011) due to perceptions of poorer physical abilities and fitness levels (Chu et al., 2020). While it is recognised that enjoyment of PA promotes the participation of autistic youth (Arnell et al., 2018; Healy et al., 2013), little is known as to what else motivates autistic youth to participate. Hence, a review of the factors that motivate (or not) autistic youth participation in PA is timely.

Several theories have been proposed to understand motivation in PA. The self-determination theory (SDT; Ryan & Deci, 2017) is one of the most widely used theories globally, providing an integrated framework for studying the social-environmental and personal factors underpinning motivation. The SDT framework (Figure 1) offers a multidimensional view of motivation, positioning motivational factors on a continuum of relative autonomy. This continuum (from none to high autonomy) includes amotivation (lack of any intention or interest to engage in the target behaviour), external regulation (behaviour is due to rewards and punishments or approval from others), introjected regulation (behaviour is driven by ego involvement, guilt and conditional self-worth), identified regulation (behaviour is enacted because of its personal importance and conscious valuing of a task), integrated regulation (behaviour/task is part of one’s identity) and intrinsic motivation (behaviour is due to feelings of enjoyment or excitement) (Deci & Ryan, 2000). A vital mini-theory embedded within SDT is the basic psychological needs theory which proposes that high-quality motivation requires the satisfaction of three basic psychological needs, those for autonomy (feeling control over one’s behaviour), competence (feeling effective in achieving personally important outcomes) and relatedness (feeling meaningfully related to and accepted by others); frustration of these needs is predictive of reduced autonomous motivation (Deci & Ryan, 2000). Socio-contextual factors (e.g. teaching styles in schools) that satisfy these needs (and hence nurture autonomous motivation) are labelled as need supportive (e.g. offering choice, acknowledging negative emotions, providing meaningful rationales for task engagement). In contrast, socio-contextual factors that frustrate these needs (and hence foster low controlled motivation) are need thwarting (e.g. using pressuring language, invoking guilt, dismissing others’ opinions and feelings; see Ntoumanis, 2023).

**Figure 1.**
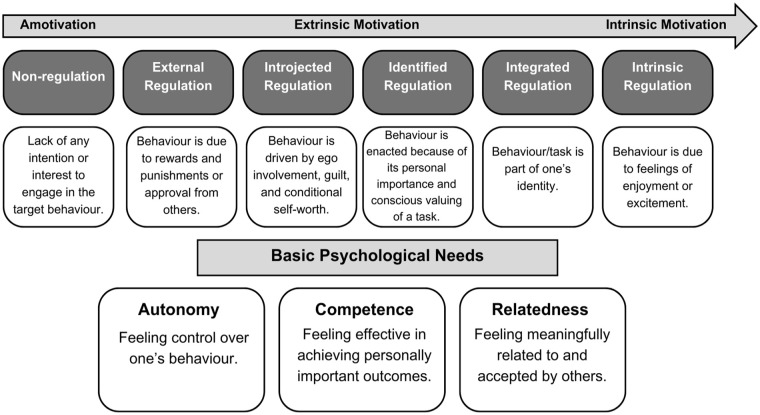
The self-determination theory framework including the continuum of relative autonomy and the basic psychological needs.

Given that motivational factors are key mediators of behaviour change and concomitant health outcomes (Ntoumanis et al., 2021; Ntoumanis & Moller, 2023), identifying those factors enabling and thwarting autistic youth’s motivation to participate in PA is necessary to supporting the health of autistic youth. Within the context of this review, the focus was on structured, as opposed to unstructured, PA. Given that SPA is the primary means through which youth obtain their PA, and as demands placed on youth differ markedly between structured and unstructured PA, it was proposed that focusing on SPA would be helpful to advancing the field. To this end, our scoping review aimed to synthesise existing literature describing the participation of autistic youth in SPA, providing a comprehensive insight into the motivations underpinning autistic youth’s engagement. This review aimed to answer the question, ‘What is known about the motivation of autistic youth to participate in SPA?’

## Method

### Protocol and registration

We aimed to scope existing literature to investigate the factors underlying autistic youths’ motivation to engage in SPA. We followed the Preferred Reporting Items for Systematic Reviews and Meta-Analyses (PRISMA)-Extension for Scoping Reviews (Tricco et al., 2018), including stakeholder consultation as recommended in guidelines of Sabiston et al. (2022) for scoping reviews. Our protocol was pre-registered with the Open Science Framework (https://doi.org/10.17605/OSF.IO/RSVM5)

### Stakeholder consultation

A stakeholder group was consulted to further explore the purpose of the review and to consider issues of readability and translation of findings during the later stages of this review (Sabiston et al., 2022). The stakeholder group comprised an autistic adolescent (*n* = 1), autistic adults (*n* = 2), parents of autistic youth (*n* = 2) and a PE teacher (*n* = 1). Stakeholders initially guided the first author on the relevance of the review and later provided input as to the validity of the results for those directly connected to the context (Sabiston et al., 2022).

### Eligibility criteria

To be eligible for inclusion in this scoping review, publications were required to (1) report data specific to school-aged, autistic participants between 5 and 18 years of age; (2) be conducted in the context of SPA; (3) be written in English; (4) be peer-reviewed and (5) measure motivational constructs, or present results with the potential to be mapped against a motivational framework. Given that the terminology relating to forms of PA can vary within the literature, we deemed studies that incorporated planned, structured activities, such as team sports, PE, structured training and other similar activities as being SPA: Quantitative, qualitative and mixed-method studies were included in the review. Theses were also included if the eligibility criteria were met. There were no restrictions on year of publication.

### Information sources and search strategy

A search strategy was developed in collaboration with a health science librarian to identify eligible articles. Electronic databases, including Medline, PsycINFO, CINAHL, SPORTDiscuss (EBSCO), ProQuest, Scopus and Web of Science were searched from their earliest records to June 2021. Researchers and the subject librarian developed an initial list of key terms and MESH headings which were grouped according to PA, autism, motivation and youth. Pilot search strings were developed using these terms and trialled in the databases. Following consultation with health science librarians, redundant terms were removed, and MESH headings exploded and were retained in the final search strategy. The MESH terms were tailored to each database. The full database search strategy is provided in the Open Science Framework (OSF) protocol (https://doi.org/10.17605/OSF.IO/RSVM5). Reference lists of included articles were also hand-searched for potentially relevant articles.

### Selection of sources of evidence

**Figure 2.**
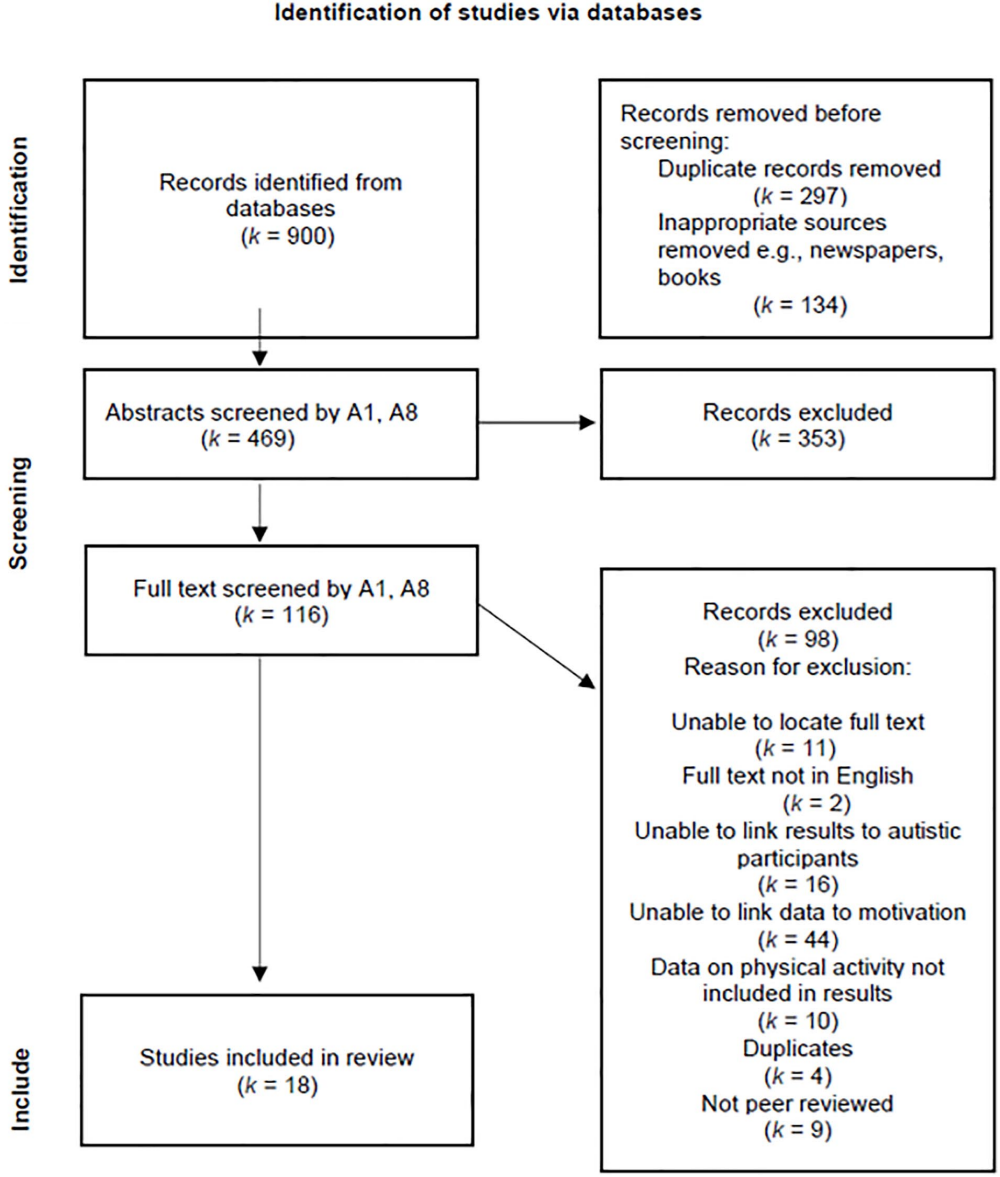
PRISMA flow diagram.

A total of 900 articles were identified through database searches. Following duplicate removal (*k* = 297) and exclusion of inappropriate sources such as newspaper articles (*k* = 134), 469 articles were screened at the abstract level. Abstract screening was performed by two authors (MLW, MHB) who independently reviewed each abstract against the inclusion and exclusion criteria. A total of 353 articles were excluded at the abstract screening level. The remaining 116 articles were then screened at the full-text level. To support full-text screening, PDFs of each article were uploaded into Research Screener. Researcher Screener is designed to be a semi-automated tool employing machine learning algorithms and data mining to streamline the screening process (Chai et al., 2021). This tool was used to organise articles and manage the screening process. This tool also uses ‘seed articles’ to train the screener to sort articles in order or relevance. For this review, three seed articles were selected and used in the tool. These seed articles were identified by authors (MLW and MHB) as meeting eligibility criteria and directly relating to influences impacting the participation of autistic youth in exercise. The seed articles were those of Arnell et al. (2018), Healy et al. (2013) and Pan et al. (2011). On the basis of these seed articles, the Research Screener application ranked the 116 articles according to their relevance. Although Researcher Screener ranked the articles to support full-text screening, all articles identified as potentially meeting inclusion criteria at the abstract screening level (*k* = 116) were independently reviewed by the two reviewers (MLW and MHB) in full-text form for inclusion and exclusion. During the screening process, both authors discussed conflicts to resolve any disagreements. If an agreement was unable to be made, a third author was consulted. Finally, 18 articles qualified for the scoping review (Figure 2).

### Charting and data items

The data items considered for extraction were decided in collaboration with three authors (MLW, SM and MHB). Data extraction obtained key information from each article including authors, year of publication, country of origin of primary university affiliated with the work, description of the study population, autism diagnosis and characteristics, study design, quantitative method of measurement (if applicable), type of participant (e.g. autistic youth parents), PA context, link to a theory of motivation, key findings and methodological quality score. Data charting was carried out independently by two authors (MLW and MHB), and key findings were then discussed in collaboration with listed authors.

### Critical appraisal of individual sources of evidence

All included articles were critically appraised as individual sources of evidence by two authors (MLW and MHB) using the Standard Quality Assessment Criteria for Evaluating Primary Research Papers from a Variety of Fields (Kmet et al., 2004). The criteria for qualitative studies comprised 10 questions, and for quantitative studies, 14 questions. A score of 0 (*no*), 1 (*partial*) or 2 (*yes*) is graded per criterion, with a total score out of 20 for qualitative designs, and 22–28 for quantitative studies (total score varies based on study designs). Results were converted to a percentage with ⩾80% considered strong quality, 70%–79% good quality, 50%–69% adequate and **<**50% limited quality (Kmet et al., 2004). Mixed-methods studies were assessed against both the quantitative and qualitative criteria.

### Synthesis of results

Data were extracted from the results section of each document and subsequently mapped according to the SDT constructs of psychological needs (autonomy, competence, relatedness) and the continuum of relative autonomy (amotivation, external, introjection, identification, integration, intrinsic motivation). A directed content analysis approach (Hsieh & Shannon, 2005) supported by the NVivo software (Zamawe, 2015) was used to map studies to the SDT framework. In this process, data derived from studies (i.e. study results) were mapped against the SDT constructs. Where studies included quantitative data, the form of sport and motivational strategy examined/employed, alongside the results, were taken into consideration when performing the mapping. For example, one study examined stationary cycling, using food as an external reward. Here, because the motivational strategy uses external reinforcement, this was mapped to the construct of external regulation. For qualitative studies, the themes derived from participant responses were examined and also mapped.

Acknowledging the unique characteristics of autism, it was anticipated that there may be factors influencing the motivation of autistic youth that did not directly align with the pre-defined SDT headings. For this reason, we conducted a second phase of analysis which utilised an inductive coding process. During this process, we sought to identify themes that may capture the unique experiences of autistic youth and the potential impact of these experiences on their motivation for SPA.

### Community involvement

Autistic youth and autistic adults were involved in the community consultation process.

## Results

### Sources of evidence

A total of 18 articles qualified for the systematic scoping review. Articles were qualitative (*k* *=* 9), quantitative (*k* *=* 7) and mixed methods (*k* *=* *2*). These included grey literature in the form of theses (*k* = 5), with the remainder being peer-reviewed articles. Key information was identified in the data-extraction process (Table 1).

**Table 1.** Characteristics of sources of evidence.

Author, year, country	Description of study population	Diagnosis and characteristics	Study design	Quantitative measurement tools	Perspective			Linked to theory of motivation				KMET score %	
					Youth	Parent	Other	Activity	Yes	No	Key findings	Qualitative	Quantitative
Alsoqairan (2019), USA	*n* = 3 Autistic youth (2 male, 1 female) 16–17 years, *m* = 16.3	Autism, non-speaking	Single case design (thesis)	BGB300 gaming cycles Polar wristband heartrate monitor	•			Stationary cycling	•		Motivation of video games can increase participation in PA		88.5
Anderson (2011), USA	*n* = 9 Autistic youth (7 male, 2 female) 6–12 years, *m* = 8 years 10 months	Autism (*DSM* 5), the ability to communicate verbally	Pilot RCT (thesis) mixed methods	LeMond Fitness G-Force RT recumbent cycle Polar E600 heart rate strap	•			Stationary cycling	•		Participants more motivated to pedal more with contingent reinforcement	92.5	65.5
Arnell et al. (2018), Sweden	*n* = 24 Autistic youth, (17 male, 7 female) 12–16 years	Autism (*DSM* 5), no co-occurring intellectual disability	Qualitative interviews, content analysis		•			SPA and PE mainstream *n* = 13, adapted education *n* = 11	•		Conceptual model of conditional participation in PA, participated when conditions met	100	
Arnell et al. (2020), Sweden	*n* = 28 Parents of autistic youth, (gender of parents NS) 33–54 years = 43	Parents N/A, child (*DSM* 5)	Qualitative interviews, content analysis			•		PE mainstream *n* = 14, adapted education *n* = 9, home school *n* = 5	•		Parents find getting adolescents to participate in PA a challenge	100	
Berends (2006), USA	*n* = 18 Parents of autistic youth, *n* = 43 CAPEs, (gender and age NS)	Parents N/A, child autism	Quantitative survey (thesis)			•	•	PE in home school		•	Parents and CAPE teachers ranked PE highly as a subject for home schooled children		70.5
Blagrave (2017), USA	*n* = 10 Autistic youth (9 male, 1 female) 10–14 years, *m* = 11.58	Autism *n* = 10 (*n* = 7 one-on-one PA specialist, *n* = 3 group setting with PA specialist)	Phenomenology using drawings and observations		•			Adapted PE class at school		•	Themes: enjoyment in participation, influence of peers and family members and the sensory experience	65	
Chu et al. (2020), Taiwan	*n* = 63 Autistic youth (males 12–18 years)	Asperger’s syndrome *n* = 20, mild autistic disorder *n* = 43, (*DSM* 5)	Cross-sectional study	BOT-2 Uniaxial GT1M Actigraph accelerometer Physical Self-Perception Profile	•			‘I can develop physical literacy’ intervention	•		PA linked to perceived competency and self-perception		97.5
Furner (2008), USA	*n* = 2 Parents of autistic child (2 female) 37 and 39 years old, *m* = 38	Parents N/A, child autism	Qualitative interviews (thesis)			•		Baseball		•	Team sports programme improved participation, increased social skills, sense of belonging	75	
Hassani et al. (2020), Iran	*n* = 30 Youth (13 male, 17 female, range 8–12 years) 15 autistic, (8 male, 7 female) *m* = 9.13, 15 non-autistic (9 male, 6 female), *m* = 9.26	autism (*DSM* 5), IQ > 70, healthy sight and hearing	Quasi-experimental trial	CAPL Grip, plank, sit and reach, 20 m PACER, body mass index (BMI), waist circumference (WC)	•			Physical activity, multiple	•		Interventions results were effective for improvement in PA		75
Healy et al. (2013), Ireland	*n* = 12 autistic youth (11 males, 1 female) 9–13 years, *m* = 11	Autism (*DSM* 5), participants in mainstream PE classes	Qualitative interviews, inductive thematic analysis		•			Mainstream PE class		•	3 Issues with mainstream PE: individual challenges, peer interactions and exclusion	100	
Jachyra et al. (2021), Canada	*n* = 10 Autistic youth (male) 12–18 years, *m* = 13.4	Autism (*DSM* 5), demonstrating verbal communication and ability to take turns in discussion	Qualitative digital stories, thematic analysis		•			Physical activity, multiple		•	Two themes: learning to be inactive and the pleasure of movement	90	
Lamb et al. (2016), UK	*n* = 5 Autistic youth (4 male, 1 female) 12–16 years, *m* = 14.4	Autism, statement of special educational need	Photo-elicitation, structured interviews (thesis)		•			Mainstream PE		•	Anticipating potential barriers can inform PE teachers	65	
Nam et al. (2020), USA	*n* = 7 Autistic youth (7 male) 5–18 years, *m* = 11.9	Autism (*DSM* 5), ability to follow simple instructions	Crossover design	LeMond Fitness G-Force RT recumbent cycle Polar E600 heart rate strap	•			Stationary cycling	•		Video extended time youth spent exercising on bike		81.5
Oriel et al. (2018), USA	*n* = 10 Autistic youth (9 males, 1 female) 10–18 years, *m* = 13.6	Autism	Pilot study, mixed methods	American College of Sports Medicine Guidelines for exercise intensity, HHR, Digital Cancellation Test (iPad) IRP-15	•			Rock climbing		•	Rock climbing a feasible activity for autistic youth	60	70.5
Pan, et al., (2011), Taiwan	*n* = 25 Autistic youth (male) m = 14.26 *n* = 75 non-autistic youth (male) *m* = 14.08	*n* = 10 Asperger’s syndrome, *n* = 15 autism (*DSM* 5)	Case-control study	Uniaxial GT1M Actigraph accelerometer MPES	•			Adapted PE class at school	•		PA and motivation less in autistic youth		91
Rosso (2016), Australia	*n* = 20 Autistic youth, *n* = 4 Down syndrome youth, (18 male, 6 female) 13–19 years, m = 15.3 *n* = 11 coaches (6 female, 5 male) 19–25 years	Autism, intellectual disability, *n* = 15 high functioning, *n* = 4 moderate functioning, *n* = 5 low functioning	Participation research	Questionnaire survey (tool not specified)	•		•	Multi-sports physical activity programme		•	Specialised sport potentially supportive of autistic youth	87.5	
Todd et al. (2010), USA	*n* = 3 Autistic youth (2 male, 1 female) 15–17 years, *m* = 16	Autism, moderate to profound disability	Multiple baseline, changing criterion	Adapted bicycle, adult tricycle Self-monitoring/goal-setting board (author created) timer/stopwatch.	•			Stationary cycling	•		Self-regulation may increase PA levels for youth	43.25	
Yessick et al. (2020), USA	*n* = 2 Autistic youth (2 male) 11–12 years, *m* = 11.6	Autistic youth in specialised school, challenging behaviours such as elopement, tantrums and aggression	Modified scrapbook study		•			Adapted PE class at school		•	Themes emerging around barriers: teacher, friends, noise	95	

*m*: mean; NS: not specified; CAPE: certified adapted physical education; *DSM* 5: Diagnostic Statistical Manual of Mental Disorders; BOT2: Bruininks–Oseretsky Test of Motor Proficiency–Second Edition; CAPL: Canadian Assessment of Physical Literacy; PACER: Progressive Aerobic Cardiovascular Endurance Run; BMI: body mass index; HHR: heart rate reserve: IRP 15: Intervention Reliability Scale; MPES: Modified Motivation in Physical Education Scale; SPA: structured physical activity; PE: physical education; RCT: randomized controlled trial.

### Characteristics of sources of evidence

Fifty percent of the included studies originated from the United States (*k* = 9). The remainder originated from Sweden (*k* = 2), Taiwan (*k* *=* 2), Australia (*k* = 1), Iran (*k* = 1), Ireland (*k* = 1), United Kingdom (*k* = 1) and Canada (*k* = 1). PE classes were the most common context of SPA and included mainstream PE classes (*k* = 5), adapted PE classes (*k* = 5) and home school PE (*k* = 2). Stationary cycling appeared in four studies. Quantitative studies (*k* = 9) employed various measurement tools, with heart rate being the most documented variable (*k* = 4). The Uniaxial GT1M Actigraph accelerometer was used in two studies to measure steps of the participants. Various survey tools were also used (*k* = 4). The majority of articles (*k* = 14) presented the viewpoint of autistic youth who were predominantly male (*n* *=* 192), with females marginally represented (*n* = 27). These numbers are representative of current autism diagnosis rates with an estimated 4:1 male/female ratio (May & Williams, 2018). Other populations represented in the included studies were parents (*k* = 3), teachers (*k* = 1) and coaches (*k* = 1). Half of the included studies (*k* = 9) did not undertake steps to confirm a formal diagnosis of autism, and the remaining studies used an autism diagnosis according to the *DSM*-5 (APA, 2013) as a criterion for inclusion. None of the included studies provided socio-demographic information on participants.

### Critical appraisal of sources of evidence

The methodological quality of several studies was strong (*k* = 10). Seven articles ranged between good and adequate while one was of limited quality. The most notable limitations were insufficient description of participants and small sample sizes. Unclear descriptions of methods and data analysis were also common limitations.

### Synthesis of results

The individual mapping of studies is displayed in Table 2.

**Table 2.** Individual data mapping results.

Manuscript author(s) and date																					
			Alsoqairan (2019)	Anderson (2011)	Arnell et al. (2018)	Arnell et al. (2020)	Berends (2006)	Blagrave (2017)	Chu et al. (2020)	Furner (2008)	Hassani et al. (2020)	Healy et al. (2013)	Jachyra et al. (2021)	Lamb et al. (2016)	Nam et al. (2020)	Oriel et al. (2018)	Pan et al. (2011)	Rosso (2016)	Todd et al. (2010)	Yessick et al. (2020)	Total
Basic psychological needs	Autonomy	Chose not to participate			•	•						•	•						•		5
		Supported	•		•	•	•											•	•		6
	Competence	Thwarted			•	•					•	•	•	•			•				7
		Supported			•				•	•	•			•		•			•		7
	Relatedness	Thwarted			•	•		•				•	•				•				6
		Supported					•	•		•		•	•					•		•	7
Continuum of relative autonomy		Amotivation			•	•							•				•				4
		External	•	•	•	•									•		•	•	•		8
		Introjection											•				•				2
		Identification			•	•		•				•					•				5
		Integration											•				•				2
		Intrinsic			•	•	•	•		•		•	•	•		•	•	•		•	12
Conditional participation model	Autism specific	Adjustment to external demands			•	•		•				•		•						•	6

#### Psychological needs

Competence was the most commonly mapped construct within included studies (*k* = 14). Perceived athletic skill and ability was reported to support and thwart competence, impacting autistic youths’ motivation to participate in SPA. Five studies documented that autistic youth who were confident that they could perform physical skills were more willing to participate in SPA (Arnell et al., 2018; Furner, 2008; Hassani et al., 2020; Lamb et al., 2016; Todd et al., 2010). As athletic demands of the PA increased, autistic youth were less inclined to participate in SPA (Arnell et al., 2018), commonly perceiving themselves to be less competent than their peers (Arnell et al., 2018; Healy et al., 2013; Pan et al., 2011). Differences in communication and ability to decode the intentions of instructions for autistic youth were also recognised as competence thwarting with one study participant describing ‘information overload . . . having a difficult time understanding, assembling, and then implementing instructions into movement patterns’ (Jachyra et al., 2021, p. 619). One study reported that competence was supported through the provision of explicit rules and direct instructions from the instructor (Lamb et al., 2016).

Relatedness was a recurrent construct across the literature (*k* = 13). Seven studies highlighted the important role of relatedness in motivating autistic youth to participate in SPA. Healy et al. (2013) noted that camaraderie and a positive rapport among classmates supported relatedness and increased autistic youths’ participation in SPA. Relatedness was also satisfied by engaging in SPA with friends, with one participant stating, ‘My friends make it [SPA] more meaningful’ (Yessick et al., 2020, p. 54). Support from family members for relatedness by facilitating the experiences of shared enjoyment, social interaction and camaraderie in SPA for autistic youth was documented in three studies (Berends, 2006; Blagrave, 2017; Jachyra et al., 2021). One of these studies identified that family SPA was used as a resource to help the autistic youth make sense of interactions with others (Jachyra et al., 2021). When engaging with neurotypical peers, autistic youth felt their differences in social communication and interactions were highlighted, leading to relatedness frustration. Arnell et al. (2018) documented the difficulties autistic youth had in adjusting socially to their peers. ‘It is hard and tiring to have to adjust to what other people say and to have activities together, then I lose interest’ (Arnell et al., 2018, p. 1797). Two articles documented the negative impact of neurotypical peers bullying autistic youth during SPA, thwarting their relatedness and negatively impacting their motivation (Healy et al., 2013; Jachyra et al., 2021). ‘It’s hard to enjoy being active when you’re always being picked on. You just learn to hate activity. Like there is no point in going to class if all I get out of it is getting hurt’ (Jachyra et al., 2021, p. 618).

Autonomy was identified in 11 articles. Four articles noted the importance of activity choice in supporting autonomy (Arnell et al., 2018, 2020; Berends, 2006; Rosso, 2016). Arnell et al. (2020) pointed to the role of parents in advocating for the modification of SPA activities in maximising autistic youths’ choice and sense of control. Autistic youth exercised their autonomy in requesting favoured games and games previously enjoyed (Rosso, 2016). Five articles noted that autistic youth, at times, chose not to participate in SPA, a choice supported by supervising adults (Arnell et al., 2018, 2020; Healy et al., 2013; Jachyra et al., 2021; Todd et al., 2010). The reasons for excluding themselves from PE lessons varied with one listing changes to routine of the lesson (Healy et al., 2013), or another stating that the wrong person appearing in the wrong place during a lesson could impact the choice to participate (Arnell et al., 2018).

#### Continuum of relative autonomy

Amotivation, being the lowest quality of motivation on the continuum of relative autonomy, refers to unwillingness to take action or participate. Four studies were mapped onto this construct, with two referring to a strong dislike of PE at school (Arnell et al., 2018; Jachyra et al., 2021). While a dislike of PE may be evident among many young people, for autistic youth, this dislike was magnified with one participant explaining that, ‘A whole day could be ruined just because of the PE . . . I almost didn’t want to go to school if we were supposed to have PE that day’ (Arnell et al., 2018, p. 1795). Another study noted that the social demands of the school day left autistic youth exhausted, with no desire or energy to participate in any further in SPA (Arnell et al., 2020).

External regulation references demonstrating motivation resulting from rewards, punishment or seeking approval of others were apparent in eight articles. Arnell et al. (2018) noted that participation in PE for some autistic youth was due to the subject being a compulsory school requirement. Four articles explicitly focussed on the role of external reinforcements in motivating autistic youth to participate in SPA. Three of these studies offered autistic youth a reward to view a chosen movie while engaging in stationary cycling, with two of these studies reporting increased time exercising for the autistic participants (Anderson, 2011; Nam et al., 2020). One study used food as an external reward, with findings in reference to its impact on increased motivation being inconclusive.

Introjection refers to behaviour driven by ego involvement, guilt and conditional self-worth. Two studies were mapped to this theme, with one reporting that autistic participants who were active acknowledged the praise they received from teachers, peers and family members (Jachyra et al., 2021). The second study was grounded in SPA and reported on introjection based on results of a self-reported motivation scale, identifying autistic youth as having lower levels of introjected motivation than their non-autistic peers (Pan et al., 2011). The reasoning behind the results on this measurement scale was not expanded on. No other references were coded to introjection within the studies.

Identification, the pursuit of an activity because of its value (i.e. identification), was mapped onto five articles within the review. Two studies described the personal importance of PA to autistic youth. Arnell et al. (2018) noted that autistic youths’ motivation to engage in SPA varied according to their beliefs of the benefits of PA. ‘I run . . . want to keep fit, I don’t want to get fat . . . I care about my weight’ (Arnell et al., 2018, p. 1796).

Integration sees a behaviour becoming a part of one’s identity, making it difficult to identify within the studies included in this review that did not report on internalised processes underlying motivation. Integration was mapped to two studies, with one of these reporting on an autistic participant’s self-identification as an active person being central to his sense of self (Jachyra et al., 2021). The second study was theoretically grounded in SDT and reported on integration based on the findings of a self-reported motivation scale, identifying autistic youth as having lower levels of integration than their non-autistic peers without providing any further information (Pan et al., 2011).

Intrinsic motivation reflecting the enjoyment and satisfaction gained from engaging in an activity emerged as a frequent construct across the included studies (*k* = 12). A variety of terms were mapped under this heading, with eight articles specifically mentioning ‘enjoyment’ as a key motivator of participating in SPA for autistic youth. Arnell et al. (2020) discussed that at times, the level of enjoyment autistic youth gained from participating in an activity overshadowed their perceived discomfort and promoted their participation. The sheer joy resulting from moving during SPA was also noted as a motivator, with one parent stating ‘physical education is a subject that makes him feel happy’ (Lamb et al., 2016, p. 11).

#### Adjustment to external demands

An additional autism-specific theme arose through the secondary inductive analysis. This theme encompassed the effect and experiences of the sensory environment on motivation *(k* = 6). While all individuals are impacted to a degree by external stimuli such as temperature and noise, this theme addresses the significant impact that differences in sensory processing can have on autistic youth (APA, 2013) and on their motivation to participate in SPA. Four articles referenced the influence of weather on SPA, noting participants had varying tolerance for weather events (Arnell et al., 2018, 2020; Blagrave, 2017; Healy et al., 2013). One article surmised that most sensory issues during SPA were a response to auditory and temperature sensitivity (Healy et al., 2013). Noise levels influenced autistic youths’ participation and enjoyment of SPA, with one participant feeling ‘lost in the chaos of all the loud noise’ (Lamb et al., 2016, p. 9).

## Discussion

This systematic scoping review explored existing research examining SPA in autistic youth, with the aim of synthesising what is known about the motivation of autistic youth to participate in SPA. This is an emerging field with many studies focussing on participation in SPA as an outcome, as opposed to the factors impacting levels of motivation for autistic youth. Nevertheless, the mapping of studies to the SDT framework allowed us to systematically explore factors influencing the motivation of autistic youth during SPA.

### Basic psychological needs

Mapping the studies to the SDT framework demonstrated that, like their neurotypical peers, satisfying the psychological needs of autonomy, relatedness and competence support autistic youths’ motivation to participate in SPA. However, findings of the reviewed studies suggest that many of these needs are not met in mainstream SPA for autistic youth. Examples of supportive SPA environments demonstrated successful adaptations to meet the needs of the autistic individual. For example, Arnell et al. (2018) identified that a ‘person-focused’ approach supported participation as opposed to a traditional ‘whole-group’ delivery. In addition, this review identified unique factors impacting motivation for autistic youth not captured by the SDT framework. These, combined with a mainstream approach to needs satisfaction, likely underly the reduced motivation to participate in SPA for autistic youth.

This review found that autistic youth are less likely to feel competent in their physical skills during SPA than their neurotypical peers. These findings reflect the high prevalence of motor difficulties in autistic individuals (Licari et al., 2020). The public nature of the SPA environment, which occurs in the context of a group or class, allows all participants to observe their peers’ ability to engage in PA and thus publicly displaying any physical challenges to the peer group. An important reason physical competency may appear low for autistic youth is their differences in communication. Autistic youth can experience difficulties interpreting instructions resulting from their challenges in filtering background sensory inputs and processing auditory information (Robertson & Baron-Cohen, 2017), impacting their competency to learn new skills and rules. Collectively, these experiences challenge autistic youths’ physical competence, reducing their levels of self-determination and willingness to engage in SPA.

Meeting the psychological need of relatedness for autistic youth proved challenging when engaging with neurotypical peers and coaches in the SPA environment. The double empathy theory suggests that a breakdown in social situations can occur when two people have differing outlooks or personal understandings of a situation (Milton, 2012). While these misunderstandings can be frustrating for neurotypical youth, they prove to be exhausting for autistic youth, thwarting the need of relatedness, ‘There was too much interaction with others and that turned out to be hard’ (Arnell et al., 2020, p. 2248). Findings in this review demonstrated that the manifestation of divergent social interactions between autistic youth and their neurodivergent peers during SPA was often exacerbated, leading to bullying and feelings of exclusion. Findings also indicated the importance of a significant other, or a small friendship group often playing the role of interpreter of the social environment for the autistic youth. Parents commonly reported playing this supportive role during SPA for their children (Arnell et al., 2020; Blagrave, 2017). The impact of personalised interactions during SPA supported the psychological need of relatedness for autistic youth (Lamb et al., 2016).

Findings from this review suggests that autistic youths’ need for autonomy varies from their neurotypical peers. For example, the requirement to choose activities freely during SPA may overwhelm some autistic youth due to their need for structure and predictability (Arnell et al., 2020). Therefore, opportunities to choose from known activities that previously resulted in positive experiences may better support autonomy for autistic youth. The choice to not participate in SPA for autistic youth was reported in the review findings. This choice was commonly supported by adults coordinating the SPA (most commonly teachers), potentially resulting in learned exclusion. Through the lens of SDT, learned exclusion may be interpreted as autonomy supportive. Further research addressing learned exclusion in autistic youth SPA participation and strategies to increase participation through autonomous motivation is required.

### Autism-specific constructs

Unique factors influencing the motivation to participate in SPA for autistic youth beyond the constructs of SDT were identified in this review. The finding that the demands of the sensory environment reduced the motivation of autistic youth to participate in SPA aligns with the sub-theme of ‘Adjustment to External Demands’ in the ‘Conditional Model of Participation’ by Arnell et al. (2018). While SDT accounts for the contextual environment and the influence this has on motivation, the theory does not account for the physical discomfort caused by the environment as experienced by many autistic youths, ‘I hate it when I get all hot and sweaty. When I get all hot my hair starts to itch uncontrollably’ (Healy et al., 2013, p. 224). Results from this review highlighted the frequent challenges autistic youth experience in regulating and processing environmental sensory input during SPA. Factors that assist autistic youth in adjusting to external demands in the SPA environment require further consideration.

The studies in this review included a variety of motivational tools and theories to engage or measure autistic youth’s participation in SPA. Some studies included the use of rewards and treats to reinforce desired behaviour, aligning with Skinner’s (1958) operant conditioning theory. While these strategies may have some impact by initially increasing autistic youths’ participation in SPA, research suggests that behavioural change resulting from external rewards or punishments is often short term (Ryan & Deci, 2017). The studies that applied SDT to assess the motivation of autistic youth relied on quantitative survey tools designed for neurotypical youth. These studies did not investigate the comprehension of the questions or the reasoning behind the choices of autistic youth who completed the survey. Considering these differences in communication for autistic youth, the effectiveness of the survey instrument requires further investigation.

The findings of this review must be interpreted in the context of several limitations.

Mapping qualitative data may introduce classification bias, with authors potentially documenting findings in support of the research aim. To reduce bias, a protocol was registered, with all reporting processes documented, and it was decided that each theme would only be mapped once per article, reducing bias of populated themes within one article. Mapping third-party qualitative data is open to misinterpretation. In undertaking the present review, multiple authors were engaged in the data extraction and mapping to the SDT framework individually and collaboratively to reduce misinterpretation of qualitative data.

### Implications

Understanding how to support the psychological needs of autistic youth has the potential to engage more youth in SPA and improve overall PA participation levels for this cohort. Further research investigating the lived experiences of autistic youth and key stakeholders in SPA will provide valuable insights into needs-supportive environments and current barriers in SPA environments that are thwarting the psychological needs of autistic youth. Future research should be guided by the prominent motivational psychology theory of SDT, the model of Conditional Participation by Arnell et al. (2018), findings of the current review and the voices of autistic youth themselves, to develop reproduceable guidelines for coaches and PE teachers to create a needs-supportive environment for autistic youth in the mainstream SPA context. This systematic scoping review provides a foundation for further examination of the facilitators and barriers supporting and thwarting motivation for participation in SPA for autistic youth.

## Conclusion

This review highlights the limited knowledge on how to support the motivation of autistic youth to participate in SPA. Findings indicated that in alignment with SDT, a needs-supportive environment impacts the motivation of autistic youth. More troublingly, findings emphasise the detrimental impact of a needs-thwarting environment on the motivation levels of autistic youth and their negative experiences in the mainstream SPA environment.

Further understanding of how to best facilitate a needs-supportive environment in SPA through the lens of autism is required. To improve SPA outcomes for autistic youth by supporting autonomous motivation, the mainstream SPA environment needs to change, not the autistic youth.
